# A histological and functional study on hippocampal formation of normal and diabetic rats

**DOI:** 10.12688/f1000research.2-151.v1

**Published:** 2013-07-09

**Authors:** Shaimaa N Amin, Sandra M Younan, Mira F Youssef, Laila A Rashed, Ibrahim Mohamady

**Affiliations:** 1 Department of Physiology, Kasr El Aini Faculty of Medicine, Cairo University, Cairo, 11451, Egypt; 2 Department of Histology, Kasr El Aini Faculty of Medicine, Cairo University, Cairo, 11451, Egypt; 3 Department of Biochemistry, Kasr El Aini Faculty of Medicine, Cairo University, Cairo, 11451, Egypt

## Abstract

**Background:** The hippocampus is a key brain area for many forms of learning and memory and is particularly sensitive to changes in glucose homeostasis.

**Aim of the work:** To investigate in experimentally induced type 1 and 2 diabetes mellitus in rat model the effect of  diabetes mellitus on cognitive functions and related markers of hippocampal synaptic plasticity, and the possible impact of blocking N-methyl-D-aspartic acid (NMDA) receptors by memantine.

**Materials and methods:** Seven rat groups were included: non-diabetic control and non-diabetic receiving memantine; type-1 diabetic groups - untreated, treated with insulin alone and treated with insulin and memantine; and type 2 diabetic groups - untreated and memantine treated. Cognitive functions were assessed by the Morris Water Maze and passive avoidance test. Biochemical analysis was done for serum glucose, serum insulin and insulin resistance. Routine histological examination was done, together with immunohistochemistry for detection of the hippocampal learning and memory plasticity marker, namely activity regulated cytoskeletal-associated protein (Arc), and the astrocytes reactivity marker, namely glial fibrillary acidic protein (GFAP).

**Results:** Both type 1 and 2 untreated diabetic groups showed significantly impaired cognitive performance compared to the non-diabetic group. Treating the type 1 diabetic group with insulin alone significantly improved cognitive performance, but significantly decreased GFAP and Arc compared to the untreated type 1 group. In addition, the type 2 diabetic groups showed a significant decrease in hippocampus GFAP and Arc compared to the non-diabetic groups. Blocking NMDA receptors by memantine significantly increased cognitive performance, GFAP and Arc in the type 1 insulin-memantine group compared to the type 1-insulin group and significantly increased Arc in the type 2-memantine group compared to the untreated type 2 diabetic group. The non-diabetic group receiving memantine was, however, significantly adversely affected.

**Conclusion:** Cognitive functions are impaired in both types of diabetes mellitus and can be improved by blockage of NMDA receptors which may spark a future therapeutic role for these receptors in diabetes-associated cognitive dysfunction.

## Introduction

Many organ systems are adversely affected by diabetes, including the brain. Patients with either type 1 or type 2 diabetes are more prone to cognitive dysfunction, including impaired memory and learning as well as Alzheimer’s disease, compared to age-matched non-diabetic subjects ^1^. Cellular mechanisms that explain how diabetes negatively influences brain functioning are still not well understood, and the most appropriatemethods to diagnose and treat cognitive dysfunctionin diabetes have not yet been defined.

The hippocampal formation (formed of the hippocampus proper, the dentate gyrus and the subiculum) is a key brain area for many forms of learning and memory, and is particularly sensitive to changes in glucose homeostasis. Analyses of behavioral performance and hippocampal synaptic plasticity in experimental models of diabetes have yielded inconsistent findings. While some studies suggested that water maze performance and passive avoidance tests as measures of synaptic plasticity were reduced ^2^, others reported that these measures were unaffected ^3^.

Astrocytes are proving critical for the survival of neurons in the central nervous system (CNS), playing a role in energy metabolism, maintenance of the blood-brain barrier, vascular reactivity, regulation of extracellular glutamate levels and protection from reactive oxygen species ^4, 5^. These cells react to neuronal damage resulting from physical or chemical insults by over expression of the glial fibrillary acidic protein (GFAP). This protects CNS cells through the uptake of excitotoxic glutamate, the production of the anti-oxidant glutathione and the neuroprotective adenosine ^6– 8^, the degradation of amyloid-beta peptides and by limiting the spread of inflammatory cells ^9, 10^. Alterations in astrocytes activity were associated with diabetes-related disturbances in the brain and levels of GFAP have been under debate ^11, 12^.

Changes in synaptic strength can occur within minutes of stimulation. For these changes to represent memory, they must persist for days and months. It is suggested that for the activity regulated and cytoskeletal associated protein (Arc), an immediate early gene may serve an important role in the transition to long-lasting forms of potentiation and hippocampal-dependent learning, memory consolidation and synaptic plasticity ^13^. N-Methyl-D-Aspartate receptors (NMDARs) are ionotropic glutamate receptors found in the CNS and it is thought that the flow of Ca ^2+^ through these receptors can cause both long-term potentiation (LTP) and long-term depression (LTD) vital for memory and learning ^14^. However, overstimulation of these receptors causes neurodegeneration and excitotoxicity ^15^.

Since the impairment of synaptic plasticity in streptozotocin (STZ)-induced diabetic rats was linked to an inappropriate level of NMDA receptor stimulation required for the induction phase of long-term-potentiation ^16^, this study aimed to investigate the effect of type 1 and type 2 diabetes on cognitive functions and hippocampal astrocyte reactivity and synaptic plasticity markers, and the impact of partial NMDARs blocking on these parameters.

## Materials and methods

### Experimental design and animals

The study protocol was approved by Physiology department committee, scientific committee and Faculty committee of Kasr Al Ainy Faculty of Medicine.

Animal experiments were performed in the Physiology Department, Kasr Al Aini Faculty of Medicine. A total of 42 male albino rats 5–6 months old obtained from Kasr Al Ainy animal house, weighing 200–250 g constituted the animal model in this study. Rats were housed each in a cage (Suzhou Suhang Technology Equipment Co., Ltd.) in a constant temperature-(22–24°C) and light-controlled room on an alternating 12:12 h light-dark cycle and had free access to food and water. Rats were fed a standard commercial pellet diet (Harlan Teklad) except groups for type 2 diabetes which were fed high fat diet (HFD) obtained by mixing 35 g of lard per 100 g of rat chow.

Rats were divided into the following groups (n=6/group):

**Table 1.** 

Control groups	Type 1 diabetes mellitus (DM) groups	Type 2 DM groups
Non-diabetic control	Untreated type 1 DM	Untreated type 2 DM
Non-diabetic memantine	Type 1 DM-insulin	Type 2 DM memantine
	Type 1-DM-insulin- memantine	

### Induction of diabetes mellitus

Type 1 diabetes was induced in rats fed on standard diet (6.5% Kcal fat) by single intraperitoneal (i.p.) injection of 65 mg/kg STZ (Biomedicals, LLC, France) dissolved in 1 ml cold citrate buffer concentration 0.1 mol PH4.8 (life technology; USA) ^17^. Type 2 diabetes was induced by feeding the rats high fat diet (HFD: 58% Kcal fat) for a period of 2 weeks followed by i.p. injection with a single lowdose of STZ 45 mg/kg. Both the low dose of STZ and the high fat diet are essential elements to induce type 2 diabetes with insulin resistance ^18^. Rats were maintained on their respective diets till the end of the study. The non-diabetic group received the i.p injection of 1 ml citrate buffer. The diagnosis of diabetes mellitus was confirmed by measuring blood glucose levels (using spectrophotometer-Beckman; USA) one week after STZ injection.

### Insulin treatment

Groups treated with insulin received (1 U/100 g) of commercial insulin (Mixtard 30/70; Novo Nordisk) once/day subcutaneous (S.C.) in the evening before the rat activity phase ^19^.

### Memantine treatment

Memantine was used in the form of commercial tablets: memantine hydrochloride (Ebixa, Lundbeck, A/S, Denmark 10 mg/tablet). Tablets were crushed, dissolved in water and the calculated dose (30 mg/kg/day) was given orally by gavage feeding for 3 weeks ^20^. Memantine treatment was started 4 weeks after induction of diabetes in corresponding groups i.e. after diagnosis of cognitive dysfunction.

### Learning and memory tests

These tests were performed 4 weeks after induction of diabetes to allow time for development of the diabetic-associated behavioural changes ^21^ and then repeated at the end of the study to assess the effect of the different treatment protocols.

### A-Passive avoidance test

The passive avoidance test is generally regarded as a measure of long-term memory, and was performed according to methods described previously ^22^. An illuminated compartment (base side; 15.5×4.5 cm, floor side; 15.5×10 cm, height 8.5 cm, 20 W) connected to a dark compartment (base side; 15.5×4.5 cm, floor side; 15.5×10 cm, height 8.5 cm) through a guillotine door was used. On habituation day, the rat was placed in the illuminated compartment and allowed to explore freely for 30 s, then the door was raised and once the rat entered the dark compartment with four paws, the door was closed and the latency to enter was recorded (from the time the door was lifted). On the following day, one learning trial was given by repeating the steps of the habituation trial and 3 seconds after the door was closed, an unavoidable scrambled electric foot shock (0.5 mA for 2 s) was delivered through the grid floor of the dark compartment and the rat was removed 30 s later to its home cage. Retention of the passive avoidance response (task) was tested 24 h later by placing the animal on the lighted compartment and measuring the latency in re-entering the dark compartment; increased escape latency to dark compartment is a good index of long-term memory.

### B-The morris water maze test

The spatial learning and memory of rats was tested according to the method of R. Morris ^23^. A Morris water maze with a submerged platform and a video tracking system (ANY-maze™ Video Tracking System; version 4.72-Stoelting Co.) were used. The Morris water maze consisted of a circular tank, (diameter: 120 cm, height: 30 cm) filled to a depth of 24 cm. The water temperature was 26°C and a 10 cm clear circular platform was submerged 1 cm below the water level in the northwest quadrant of the maze.

***Cue discrimination.*** Using the protocol seen in ^24^ and ^25^. A visible platform test was performed to exclude drug or experimental manipulation-induced changes in visual acuity. The video tracker system was not used and only a stop watch was used in this test. Habituation to the pool was done by permitting the rats to swim freely for 30 seconds and giving them four trials (from four different directions) to climb to the platform that had been extended 1 cm above the water level. The rats then had 15 trials of cue training in 3 block intervals, each including five trials; the intervals (intertrials and interblock) were approximately 10 minutes. During this stage we didn't provide the rats with cues except for the platform.

***Spatial discrimination.*** During spatial discrimination, the hidden platform was placed 1.5 cm below the water level changing the area of the pool from that used during cue discrimination training. We added powered milk to make the pool water opaque, rendering the platform ‘invisible’. The platform location had been fixed relative to the distal cues. Rats had trained in eighteen trials in the form of six blocks (three trials per block) and the intertrial intervals were about 10 minutes and after every trial we stirred the water to avoid the effect of odor trails as unwanted cues. Rats were allowed to start swimming in each trial from one of four locations (north, south, east, and west); the choice of the location was random for each rat and each trial. The rat should escape to the platform within 60 seconds and if that didn't occur we guided them gently toward the hidden platform where they remained for 10 seconds. The rats were dried with a towel and returned to their cage after every trial. The parameters recorded in these training blocks were: latency to reach the platform, distance traveled in the maze till reaching the platform and proximity (% of time spent within the quadrant where the platform was placed).

***Probe trial.*** In the probe trial (the immediate probe trial) we removed the platform from the swimming pool and allowed the rat to swim for 60 seconds. The probe trial was given after the fifth training block and the rats then had the sixth block of training that was not included in cognitive assessment. In order to assess 24-hour retention, rats were given another probe trial 24 hours later (the platform was removed from the pool).

### Biochemical analysis

At the end of experimental period rats were anesthetized with ether, blood samples were collected from retro-orbital venous sinus, fasting serum glucose was measured using oxidase-peroxidase method ^26^ and fasting serum insulin was analyzed using enzyme-linked immunosorbent assay (ELISA) (DRG diagnostics, Germany) according to the manufacturer’s instructions. To estimate insulin resistance, the homeostasis model assessment for insulin resistance (HOMA-IR: insulin resistance index) was calculated ^27^. HOMA-IR is an indirect method for the assessment of insulin resistance. It depends on relationship between fasting plasma glucose and insulin based on a mathematical model. HOMA-IR was calculated using the following equation ^28^:

HOMA-IR = Fasting glucose (mg/dl) × Fasting insulin (uU/ml)/450

### Histological staining

***Brain sectioning and staining.*** The rats were anesthetized with ether followed by quick cervical dislocation. After death confirmation by lack of pulse, breathing, corneal reflex and response to firm toe pinch, inability to hear respiratory sounds and heartbeat by use of a stethoscope then we performed decapitation followed by harvesting of brain tissues which were placed in 10% formaldhyde for 2 hours ^29^. The brains were removed and placed in a new formaldehyde solution for 24 hours before being dehydrated using ethanol (70% for 24 h, 90% for 1 h and 100% for 1 h) then cleaned in xylene and embedded in paraffin. Coronal sections were cut with a microtome (Leica RM 2025, Germany) at 5 µm thicknesses, mounted on glass slides and stained with the routine hematoxylin and eosin technique ^30^. Examination of slides, photography and morphometric studies were done at Histology Department, Kasr El-Aini Faculty of Medicine.

***Immunohistochemical techniques.*** Serial brain sections cut at 5 µm thickness were mounted on positively charged glass slides (SuperFrost Plus® slides, MENZEL-GLÄSER) for immunohistochemical staining using primary antibodies: glial fibrillary acidic protein (GFAP) (ThermoScientific, USA, ready to use, 7 ml), and activity regulated-cytoskeletal associated protein (Arc) (Lifespan Biosciences, Seattle, WA-µl diluted 1:500) together with a secondary "Ultravision detection system" (ThermoScientific, USA, catalog no TP-015-HD). Sections were deparaffinised and hydrated in graded descending concentrations of alcohol, then incubated with hydrogen peroxide blocking solution (3%; ThermoScientific, USA) for 15 mins. Incubation was done in humid chambers at room temperature, and slides were continuously kept wet starting from this step onwards. Slides were washed twice in phosphate buffer (0.15 mol/L NaCl, pH 7.0; Dako; Denmark) incubated with pepsin digestive enzyme-one packet dissolved in 500 ml of 0.2 N Hcl (Dako; Denmark) and washed 4 times in buffer. Ultra V block (ThermoScientific, USA) was applied and incubated for 5 mins. Primary antibodies were then applied on the serial sections and each was incubated for 30 mins. Sections were then washed and biotinylated goat antipolyvalent antibody (secondary antibody) was applied for 10 mins, washed, then followed by streptavidin peroxidase for 10 mins and washed after. To develop color reaction, one drop of DAB Plus chromogen was added to 2 ml of DAB Plus substrate, mixed and applied on tissues for 5–15 mins. Sections were then counterstained with Mayer’s hematoxylin. A coverslip was applied using mounting media. A positive reaction appeared as brown color ^31^.

***Quantitative morphometric study.*** Ten high-power fields were measured in each of the serial sections in the different studied groups. Area % was measured for GFAP and Arc. The data were obtained by using Leica QWin 500 image analyzer computer system (England). The image analyzer consists of an Olympus microscope, a colored video camera, colored monitor and a hard disc of a Leica IBM personal computer connected to the microscope and controlled by Leica QWin 500 software. Data were statistically described in terms of mean and standard deviation (mean±SD) for area %. Photography was performed using a Panasonic wv. GP 210 camera connected to the Olympus microscope (U-CMAD3-Japan) and Olympus camera for measurments (C3040-ADU, Japan) connected to the computer.

## Statistics

The results were analyzed using SPSS computer software package, version 16 (Statistical Package for the Social Science; SPSS Inc., Chicago, IL, USA). Data were presented as mean±SD. Comparison of quantitative variables between the studied groups was done using analysis of variance (ANOVA) test with the Bonferroni post Hoc test or Kruskal Wallis test with Wilcoxon signed rank test depending on the result of the Shapiro-Wilk test for normality of distribution which determined if data was parametric or non-parametric. Results were considered statistically significant at *p*≤0.05 ^32^.

## Results

### Metabolic parameters in the different studied groups

As revealed from Table 2, administration of memantine to non-diabetic rats significantly increased serum glucose compared to non-diabetic group. No statistical difference was observed in serum insulin and HOMA-IR between both groups.

**Table 2.** Metabolic parameters in the different groups.

Measured parameters	Non-diabetic	Non-diabetic- memantine	Untreated type 1 diabetic	Type 1 diabetic- insulin	Type 1 diabetic- insulin- memantine	Untreated type 2 diabetic	Type 2 diabetic- memantine
**Serum glucose** **(mg/dl)**	76.6±6.2	113.33±1.36*	295.3±9.6*	112.33±3*#	103±5.8*+@	280.17±8.15*	228.50±7.17+$
**Serum insulin** **(μU/ml)**	4.1±0.76	4.2±0.50	2.2±0.55*	5±0.53#	5.31±0.62	11.6±1.11*	9.66±0.56+$
**HOMA-IR**	0.77±0.16	1.17±0.15	1.64±0.37*	1.41±0.27*#	1.37±0.27+	8.17±0.48*	5.53±0.28+$

*: significant compared to non-diabetic group.

+: significant compared to non-diabetic-memantine group.

#: significant compared to untreated type 1 diabetic group.

@: significant compared to type-1 DM-insulin group.

$: significant compared to untreated type 2 DM group.

As expected, induction of type 1 diabetes mellitus significantly increased serum glucose level and HOMA-IR, and significantly decreased serum insulin in the untreated type 1 DM group ( *p*<0.05) compared to non-diabetic group.

The type 1 DM group treated with insulin or with insulin-memantine still had significantly increased serum glucose and HOMA-IR compared to the non-diabetic group and non-diabetic-memantine group, respectively. The type 1 insulin treated group showed a significantly decreased serum glucose with a significantly increased serum insulin and HOMA-IR compared to the untreated type 1 diabetic group ( Table 2, *p*<0.05). The type 1 DM group treated with both insulin and memantine showed a significantly decreased serum glucose ( *p*<0.05) with no statistical difference in the serum insulin and insulin resistance index compared to type 1 DM group treated with insulin alone ( *p*>0.05).

Induction of type 2 diabetes significantly increased serum glucose and insulin levels as well as HOMA-IR in untreated type 2 DM group compared to the non-diabetic group. Type 2 DM group treated with memantine showed a significant decrease in serum glucose and insulin and HOMA-IR compared to untreated type 2 DM group, although these parameters were still significantly increased compared to the non-diabetic-memantine group ( Table 2, *p*<0.05). These results indicate that while memantine can improve serum glucose in both types of diabetes mellitus, it has no beneficial effect in non-diabetic rats.

### Effect of diabetes and memantine on cognitive functions

**Figure 1.**
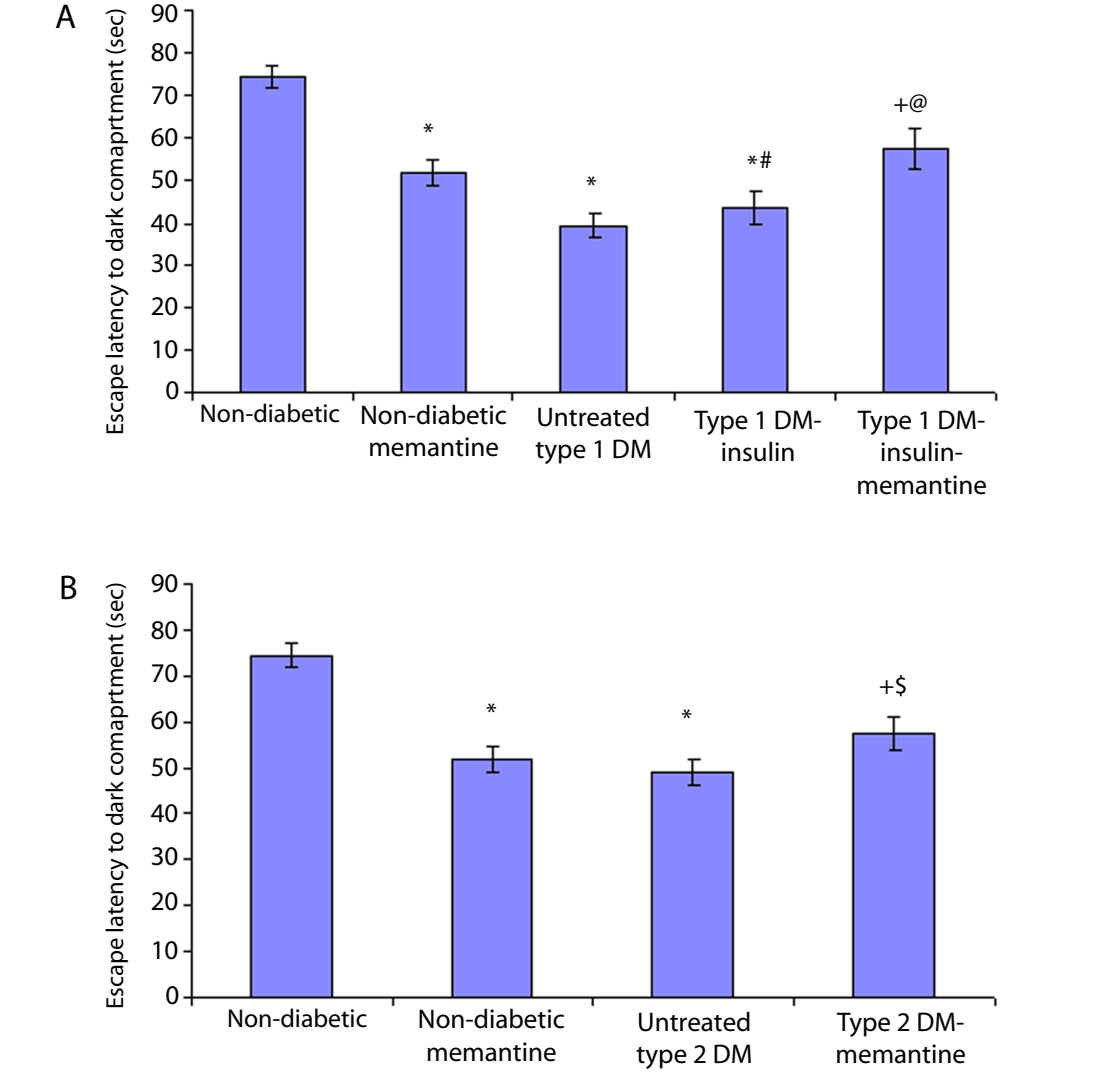
Escape latency recorded during passive avoidance test. Latency to enter the dark compartment in type 1 diabetic ( **A**) and type 2 diabetic ( **B**) subgroups compared to non-diabetic subgroups. *: significant compared to non-diabetic group, +: significant compared to non-diabetic-memantine group, #: significant compared to untreated type 1 diabetic group, @: significant compared to type 1-insulin group, $: significant compared to untreated type 2 diabetic group at *p*<0.05.

**Figure 2.**
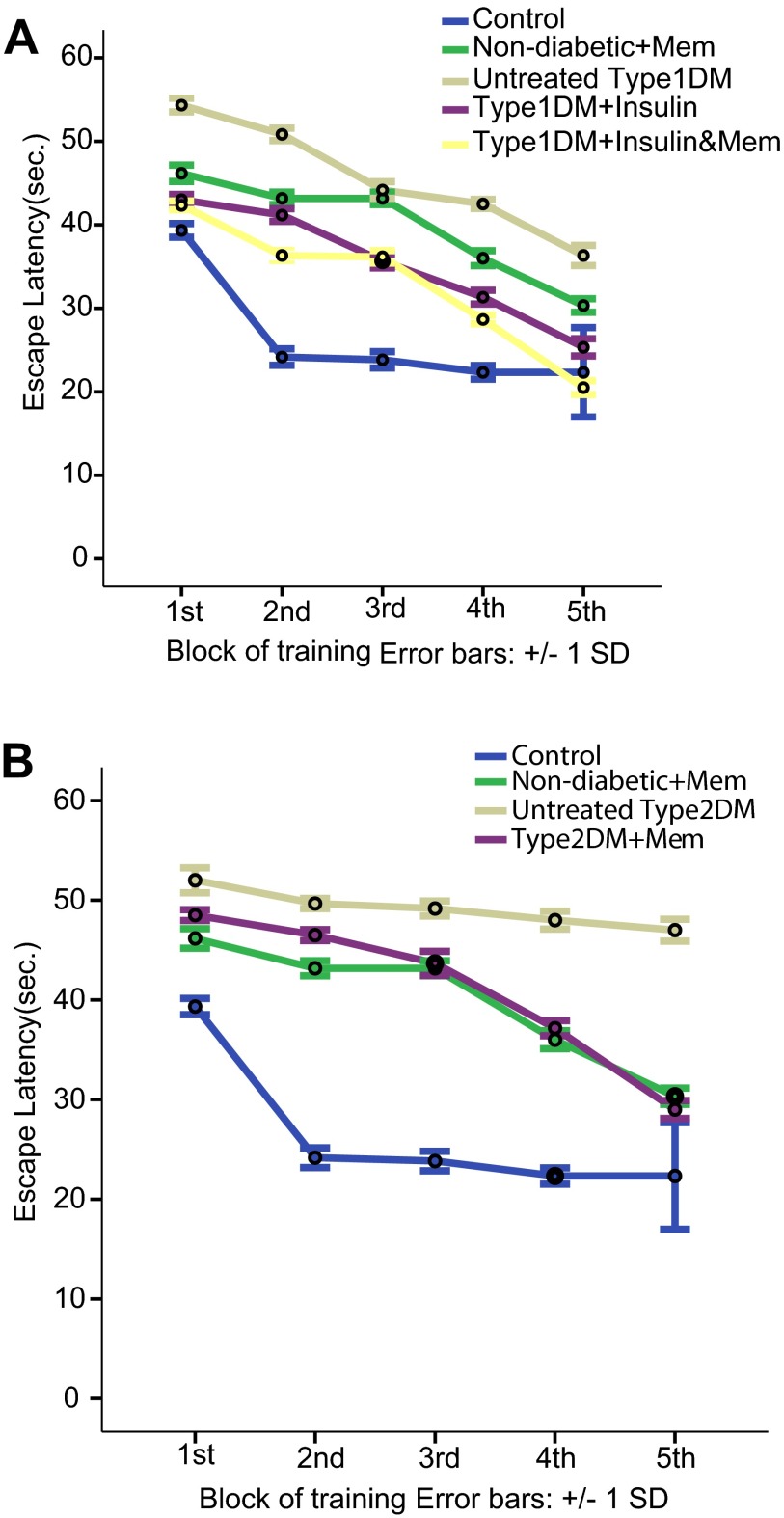
Latency to reach the hidden platform during training blocks of Morris water maze in the different groups. **A**: escape latency to hidden platform in type 1 diabetic subgroups compared to non-diabetic groups. **B**: escape latency to hidden platform in type 2 subgroups compared to non-diabetic groups. Data are presented as mean±SD (n=6/group).

**Figure 3.**
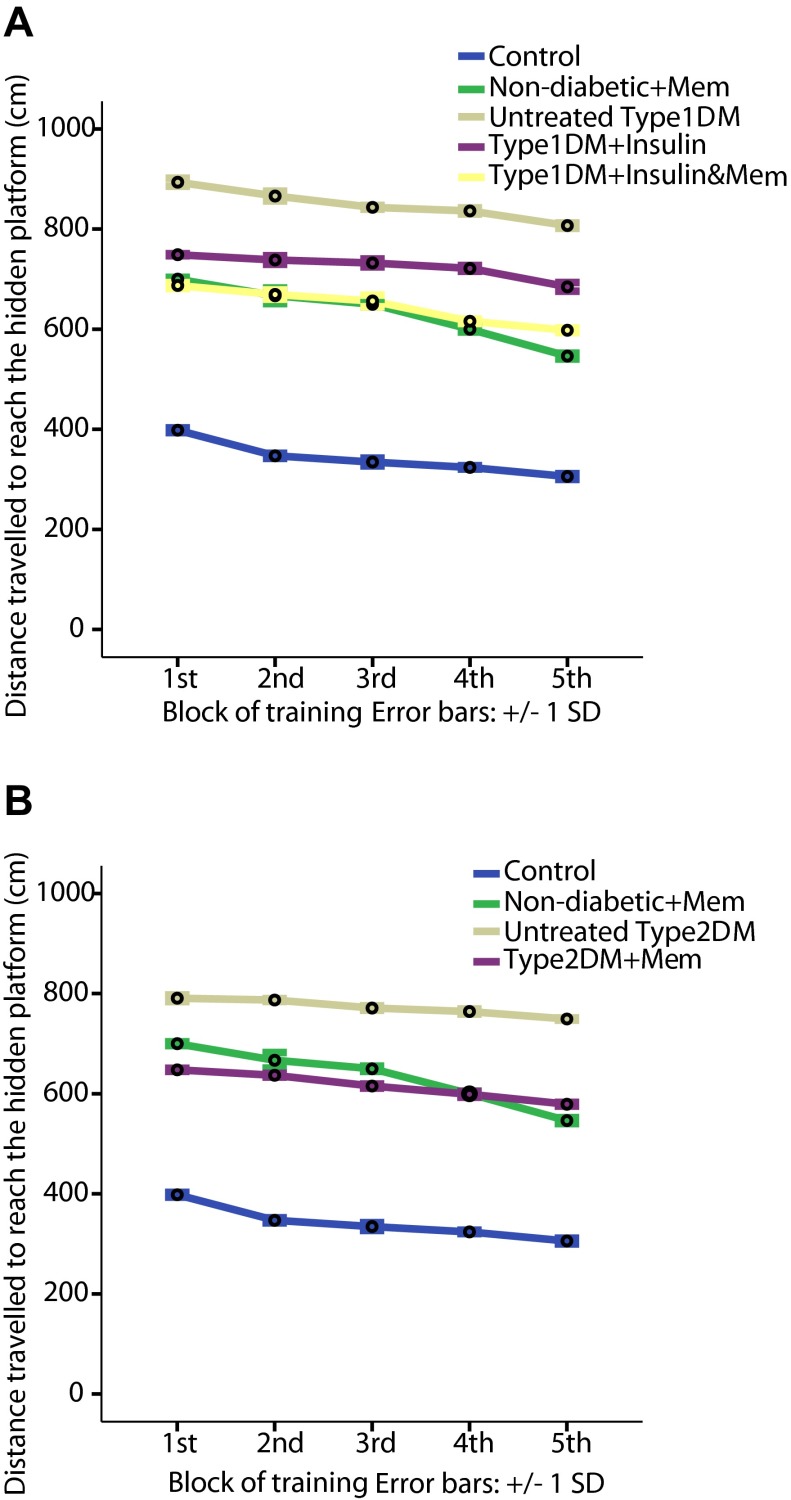
Distance travelled to reach the hidden platform during training blocks of Morris water maze in the different groups. **A**: distance travelled to hidden platform by type 1 diabetic subgroups compared to non-diabetic groups. **B**: distance travelled to hidden platform by type 2 subgroups compared to non-diabetic groups. Data are presented as mean±SD (n=6/group).

**Figure 4.**
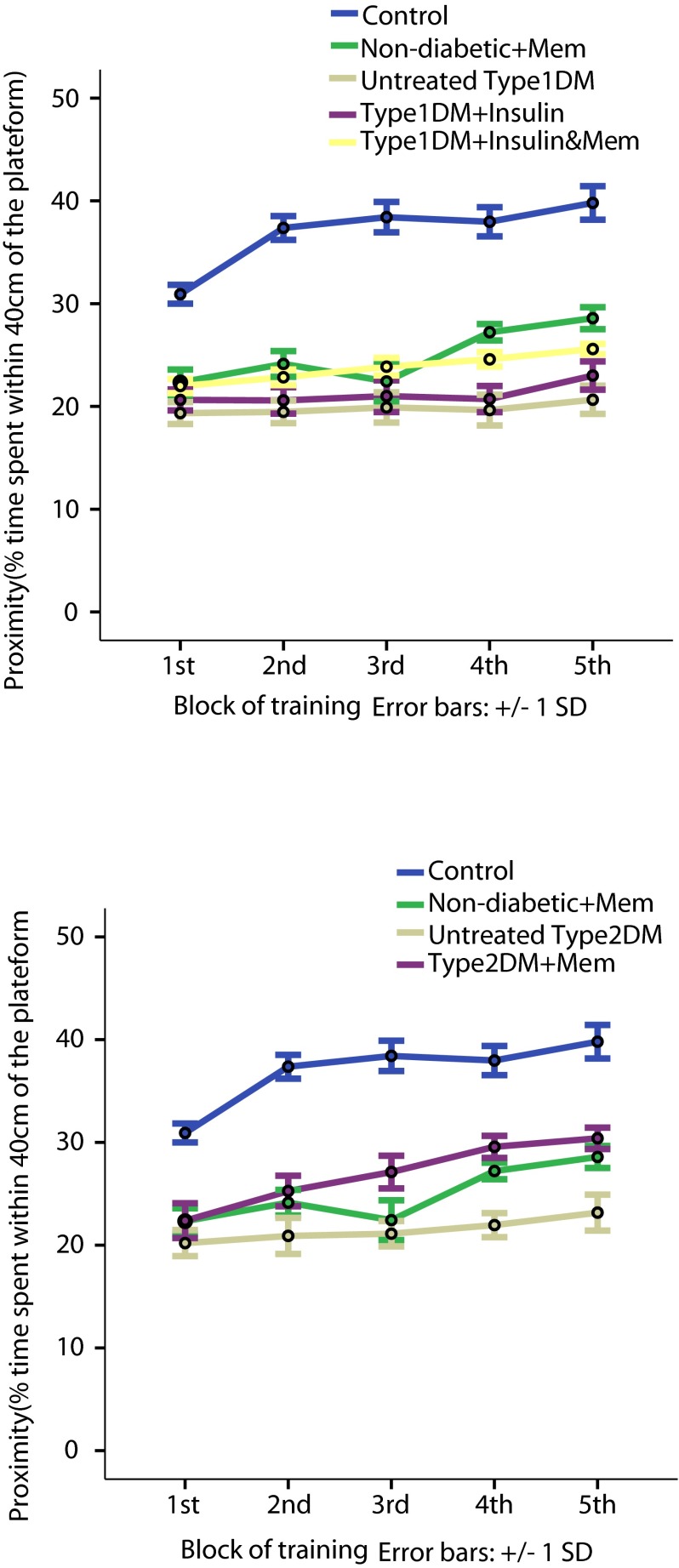
Proximity during training blocks of Morris water maze in the different groups. Proximity (% time spent within 40 cm of the platform) in type 1 ( **A**) and in type 2 ( **B**) diabetic subgroups compared to non-diabetic groups. Data are presented as mean±SD (n=6/group).

**Figure 5.**
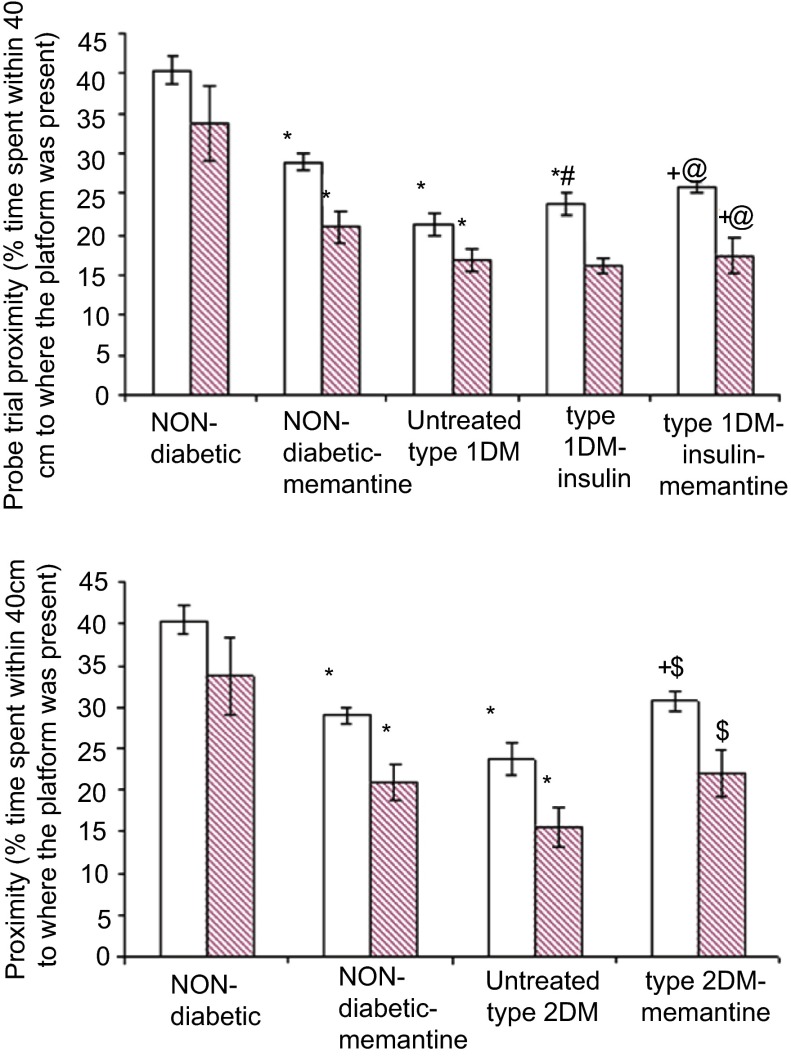
Proximity during immediate and 24 h probe trials in Morris water maze of the different groups. Proximity (% time spent within 40 cm to where the platform was previously present) in type 1 ( **A**) and in type 2 ( **B**) diabetic subgroups compared to non-diabetic group during immediate (open bars) and 24 h (filled bars) probe trials. *: significant compared to non-diabetic group, +: significant compared to non-diabetic-memantine group, #: significant compared to untreated type 1 diabetic group, @: significant compared to type 1-insulin group, $: significant compared to untreated type 2 diabetic group at *p*<0.05. Data are presented as mean±SD (n=6/group).

Results indicate that both types of diabetes induced deficiency in learning and spatial memory in rats during the passive avoidance and Morris water maze tests. Untreated type 1 and 2 diabetic groups compared to the non-diabetic group exhibited a significant decrease in the escape latency to the dark compartment in the passive avoidance test ( Figure 1A&B, *p*<0.05), a significant increase in the escape latency and the travelled distance to hidden platform ( Figure 2A&B and Figure 3A&B, *p*<0.05) and a significant decrease in proximity as a measure of the % time spent within 40 cm of the platform ( Figure 4A&B, *p*<0.05) in all Morris maze training blocks. Also, a significant decrease was observed in the proximity of both the untreated diabetic groups during immediate and 24 h probe trials compared to the non-diabetic group ( Figure 5A&B, *p*<0.05).

The type 1 diabetic group treated with insulin also showed a significant impairment in all performed tests compared to the non-diabetic group ( *p*<0.05), and a partial improvement of the cognitive functions compared to the untreated type 1 diabetic group. Compared to the non-diabetic group, the insulin-treated group showed a significant decrease in the escape latency to the dark compartment in the passive avoidance test ( Figure 1A, *p*<0.05), a significant increase in the escape latency and the travelled distance to the hidden platform ( Figure 2A and Figure 3A, *p*<0.05), and a significant decrease in proximity observed during training and during immediate and 24 h probe trials ( Figure 4A and Figure 5A, *p*<0.05) of the Morris water maze.

Compared to the untreated type 1 diabetic group, the insulin-treated type 1 group showed a significant increase in the escape latency to the dark compartment of the passive avoidance test ( Figure 1A), a significant decrease in the escape latency ( Figure 2A) and distance travelled ( Figure 3A) in all blocks of training and a significant increase in proximity only during the 1 ^st^ and 5 ^th^ blocks of training and immediate probe trial during the Morris water maze test ( Figure 4A and Figure 5A, *p*<0.05).

Treating the type 1 diabetic group with both insulin and memantine had a better impact on learning and spatial memory compared to insulin treatment alone. A significant increase was observed in the escape latency to the dark compartment of the passive avoidance test ( Figure 1A) compared to the non-diabetic memantine- and insulin-treated type 1 groups. Also a significant decrease in escape latency to the hidden platform ( Figure 2A) was revealed compared to the non-diabetic-memantine group in all training blocks and in the 4 ^th^ and 5 ^th^ training blocks compared to the type 1 diabetic-insulin group. Distance travelled in the Morris water maze test by the type 1-insulin-memantine group was significantly decreased in all blocks compared to the type 1 DM-insulin group ( Figure 3A) and in the 1 ^st^, 4 ^th^ and 5 ^th^ block of training compared to the non-diabetic-memantine group indicating a beneficial effect of memantine over the insulin treatment alone.

Proximity in the type 1 insulin-memantine group was significantly increased in all blocks of training ( Figure 4A) and in immediate and 24 h probe trials ( Figure 5A) compared to that of the type 1 diabetic-insulin group. However, this was still significantly decreased in training blocks 4 and 5 and in the immediate and 24 h probe trials compared to non-diabetic-memantine group ( Figure 4A and Figure 5A, *p*<0.05).

Treating the type 2 diabetic group with memantine significantly increased escape latency to the dark compartment of the passive avoidance test ( Figure 1A) compared to the non-diabetic-memantine and untreated type 2 diabetic groups. Also, it significantly decreased the escape latency to the hidden platform ( Figure 2B) compared to the untreated type 2 diabetic group in all Morris water maze training blocks, although this still significantly increased compared to non-diabetic-memantine in the 1 ^st^ 4 blocks and only significantly decreased in the 5 ^th^ training block.

Distance travelled in the Morris water maze was significantly decreased in the type 2-memantine group compared to untreated type 2 group in all training blocks, and to the non-diabetic-memantine group in the first three blocks with a statistically insignificant difference in the last two blocks, which indicated a positive effect of memantine on learning and spatial memory.

Proximity was significantly increased in the type 2 memantine group in the last four training blocks and in immediate and 24 h probe trials compared to the untreated type 2 DM. Moreover; proximity was significantly increased in the last three training blocks and the immediate probe trial in the type 2 memantine group compared to the non-diabetic-memantine group. These results suggest that memantine may consolidate the long-term memory in type 2 diabetes.

Administration of memantine to the non-diabetic group had less beneficial effects with a significant decrease in the escape latency to the dark compartment in the passive avoidance test ( Figure 1) and a significant increase in the escape latency and distance travelled to the hidden platform during all training blocks of the Morris water maze test compared to the non-diabetic group ( Figure 2 and Figure 3, *p*<0.05). Proximity was also significantly reduced in all training blocks and in the immediate and 24 h probe trials compared to the non-diabetic group ( Figure 4 and Figure 5, *p*<0.05), which suggest that memantine has no role in learning and memory consolidation in the non-diabetic state.

## Histological results

### Hematoxylin and eosin stained sections

**Figure 6.**
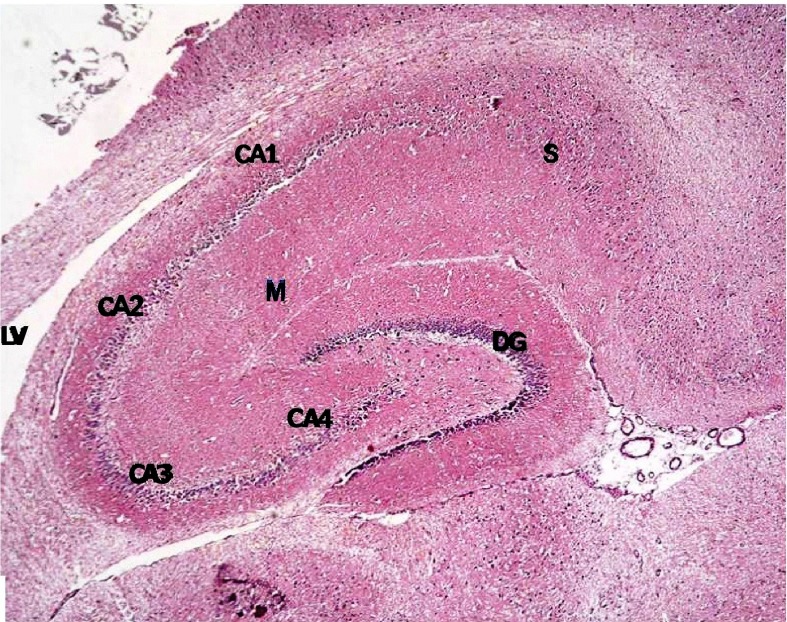
Section from non-diabetic control showing the different areas of the hippocampal formation where the hippocampus proper is formed of the Cornu Ammonis (CA) as CA1, CA2, CA3 & CA4 regions, and is continued as subiculum (S). Dentate gyrus (DG) is seen surrounding CA4 by its upper & lower limbs. Note lateral ventricle (LV) related to CA1 & CA2. M denotes molecular layer inside concavity of CA and of DG. (H & E ×40).

Histological examination of hematoxylin and eosin stained sections revealed the characteristic areas of hippocampal formation. These are the hippocampus proper, dentate gyrus and subiculum ( Figure 6).

**Figure 7.**
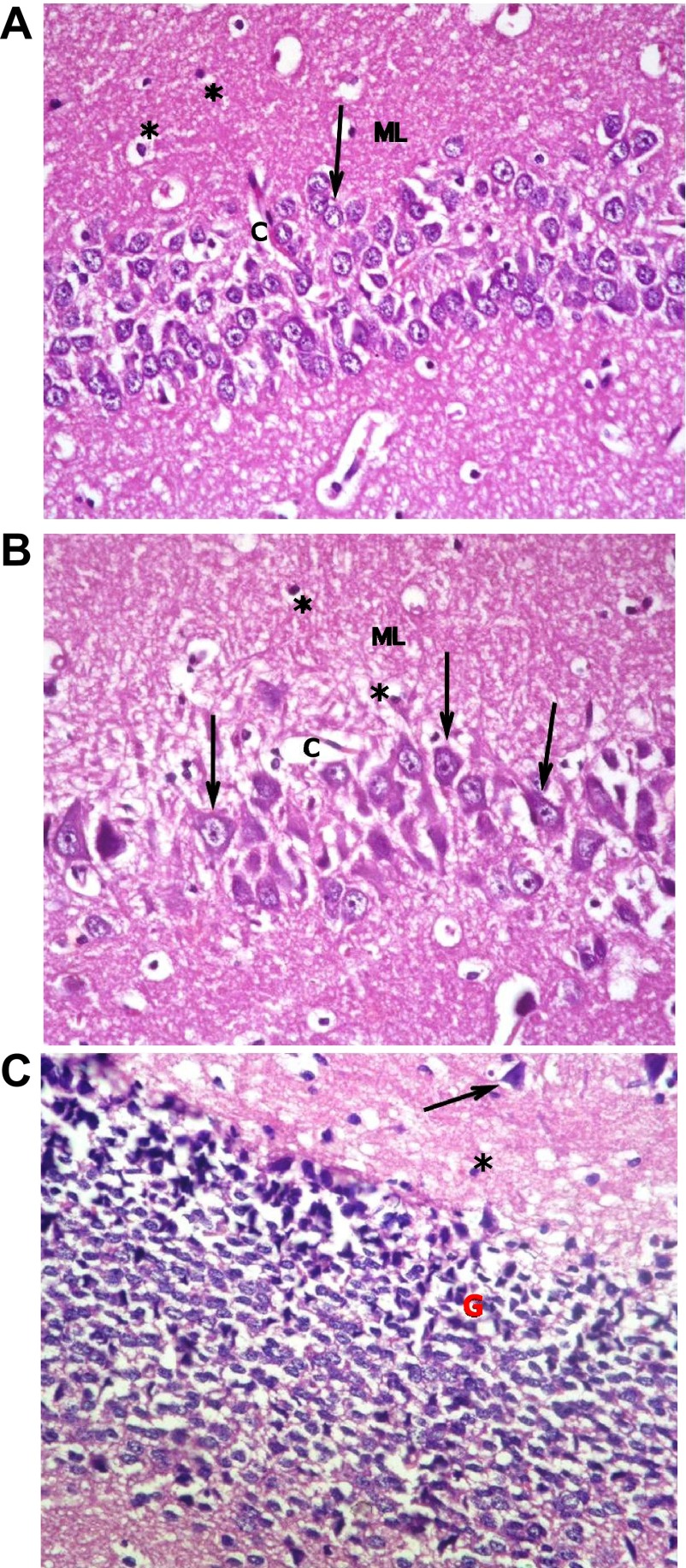
( **a**): Section from non-diabetic control showing 5–6 compact layers of small pyramidal cells of CA1 region, most with vesicular nuclei; ( **b**): shows few layers of large pyramidal cells in CA3 region, also with vesicular nuclei (arrows). Molecular layer (ML) shows many glial cells (*) among neuronal processes. ( **c**): shows layers of compact granular cells with dark nuclei in dentate gyrus G. Molecular layer shows glial cells (*) as well as pyramidal cells (↑) (H & E ×400).

The hippocampus proper is formed of Cornu Ammonis CA1 and CA2 formed of zone of small pyramidal cells, CA3 and CA4 formed of zone of large pyramidal cells. CA4 projects into concavity of dentate gyrus that is formed of small granule cells. Subiculum is outward continuation of CA1 region. Areas in between compact zones of cells comprise the molecular layer which consists of neuronal processes/(axons and dendrites), glial cells, and scattered nerve cells ( Figure 7).

**Figure 8.**
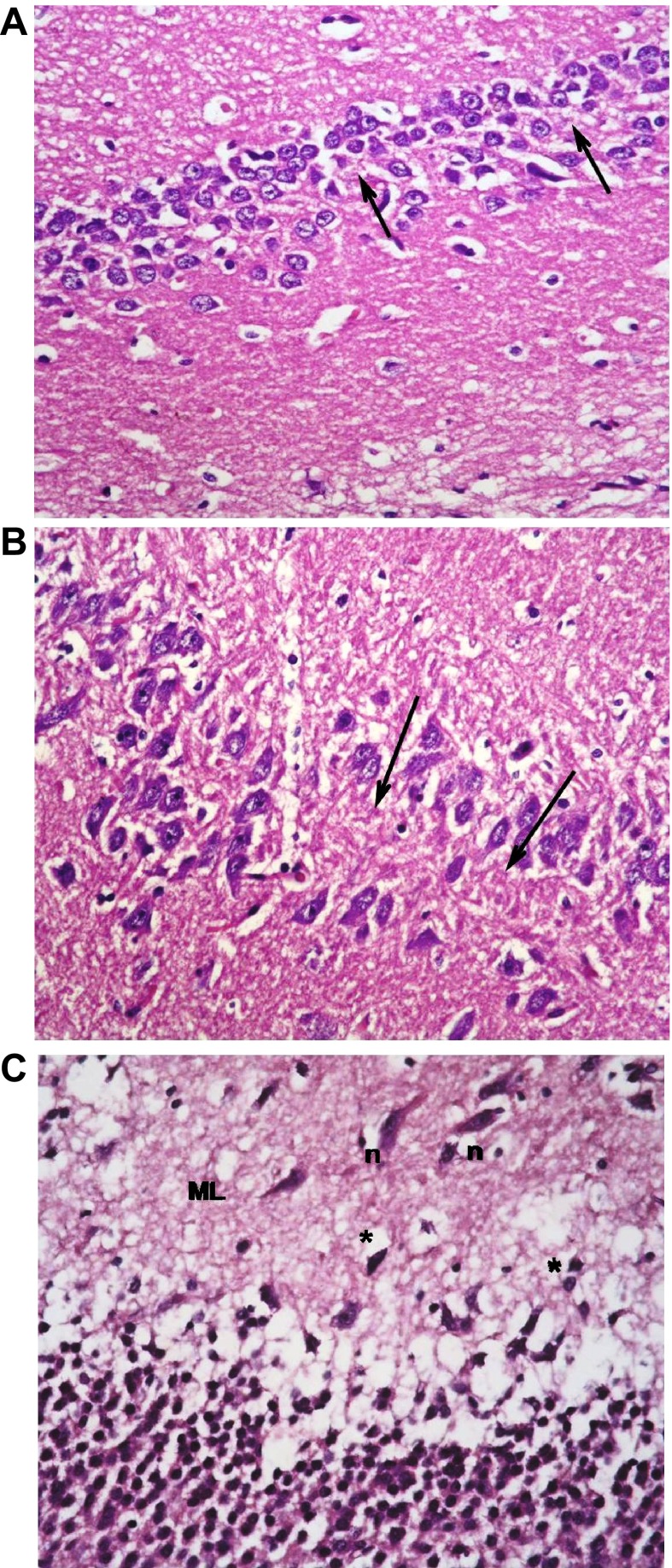
Section from non-diabetic group receiving memantine alone showing: ( **a**): decreased thickness of layer of small pyramidal cells of CA1 to reach 2 layers in some areas (↑); and ( **b**): more marked affection of large pyramidal cells of CA3 where areas are devoid of cells (↑). Cells have vesicular nuclei. ( **c**): granular cells show marked retraction of processes with vacuolations, and molecular layer (ML) shows enlarged neurons (n) and enlarged glial cells (*). (H & E ×400).

Sections from non-diabetic groups receiving memantine alone were shown to be markedly affected by the treatment showing decreased thickness of the small pyramidal cell layers, decreased thickness and areas of cell loss of the large pyramidal layers, with retraction of processes and vacuolation of granular cells. The molecular layer exhibited enlarged pyramidal cells ( Figure 8).

**Figure 9.**
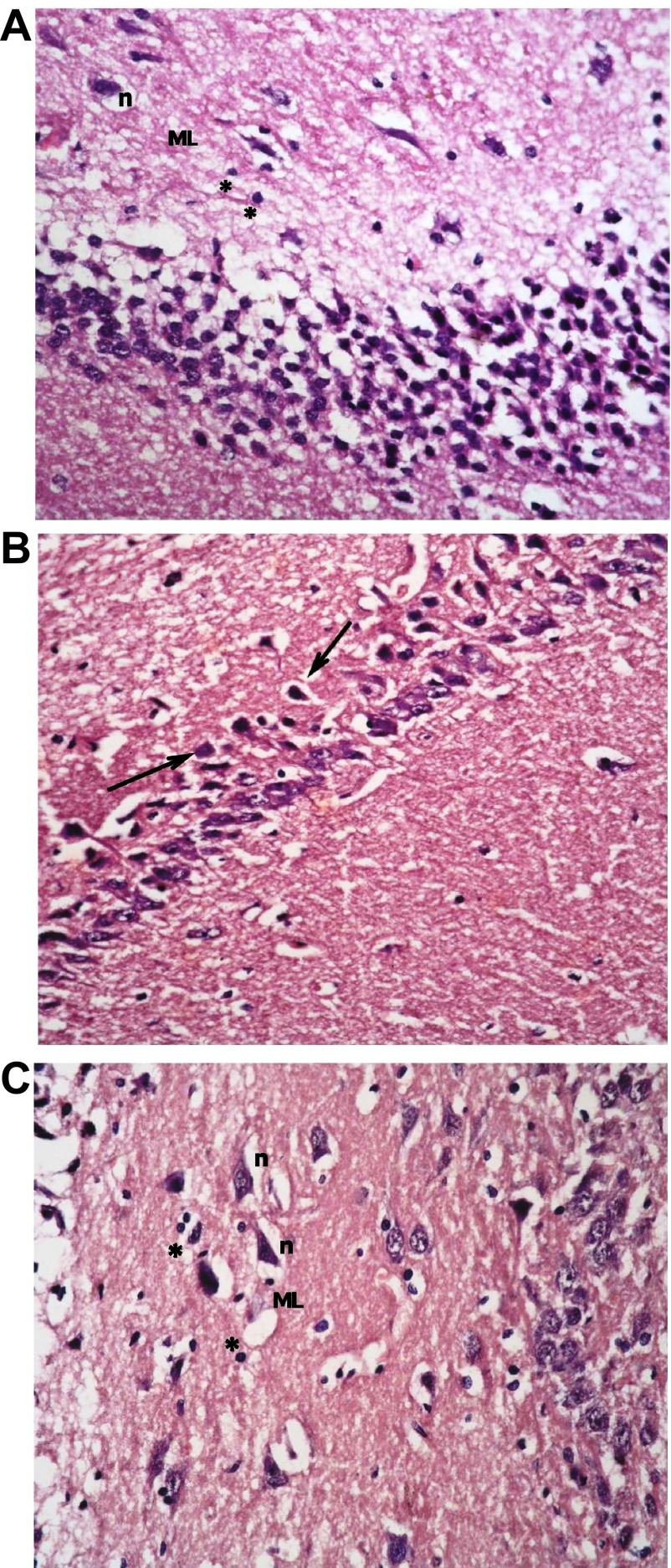
The untreated type 1 diabetic group shows ( **a**): disorganization and areas of cell loss of small pyramidal cells; some having pale nuclei and others dark. Note also clumping of neuronal processes. ( **b**): marked shrinkage in size of large pyramidal cells, affecting outer layer more, with darkened nuclei (↑). ( **c**): Granular cell layers also showed marked vacuolations. Molecular layer (ML) shows marked enlargement of neurons (n) and of glial cells (*).

The type 1 DM group that was left untreated showed marked changes in all regions in the form of disorganization and cell loss of small pyramidal cells, some of which had pale nuclei while others were dark. There was also marked shrinkage in the size of the large pyramidal cells, the outer layer was more affected, with darkened nuclei. The granular layer also showed marked vacuolation, while the molecular layer exhibited enlarged neurons and excess glial cells ( Figure 9).

**Figure 10.**
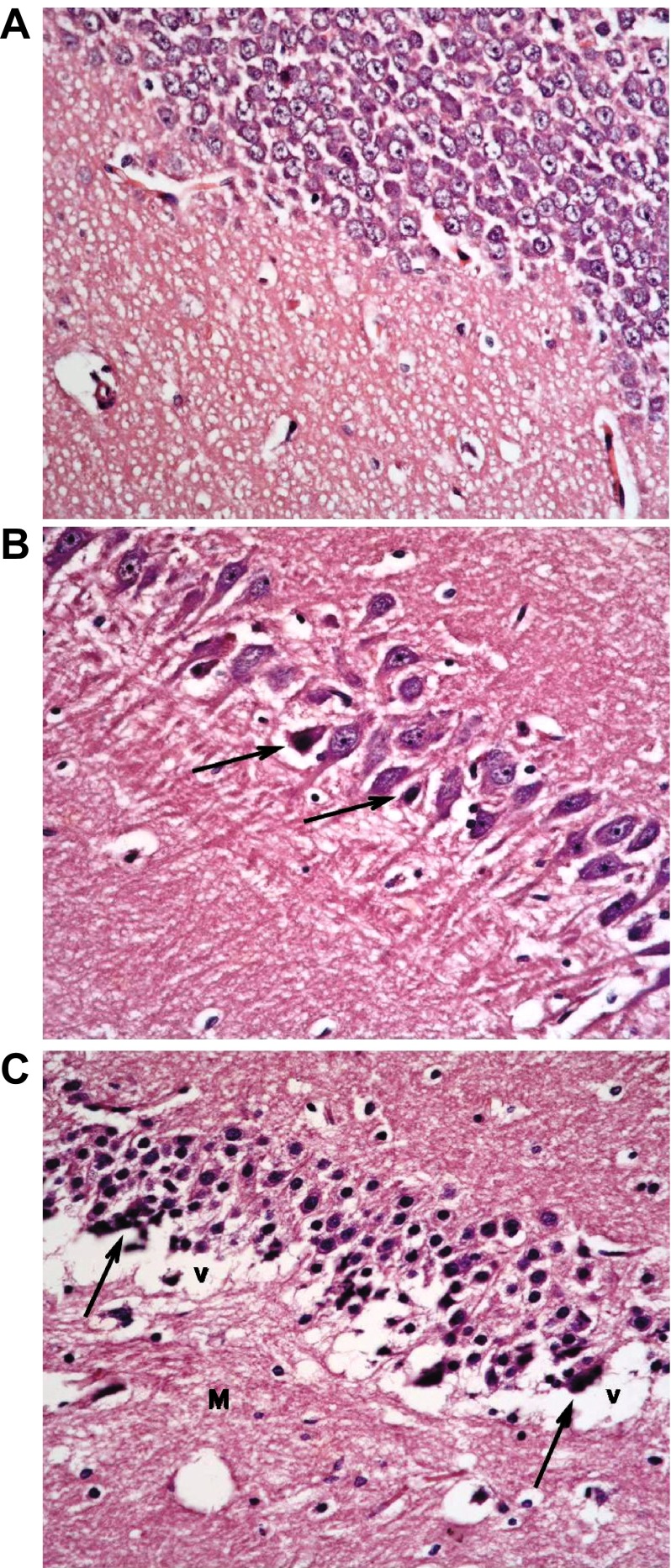
The type 1 diabetic group treated with insulin only shows ( **a**): preservation of small pyramidal cells; but with ( **b**): marked apoptosis of large pyramidal cells (↑) and ( **c**): marked disorganization, vacuolation (V) and decreased population of granular cells. Molecular layer (M) mostly shows normal cells & fibres. (H & E ×400).

Treatment with insulin alone caused improvement in the form of preservation of small pyramidal cells and markedly decreased apoptosis of large cells. Vacuolations, however, persisted in granular cells together with overall marked disorganization ( Figure 10).

**Figure 11.**
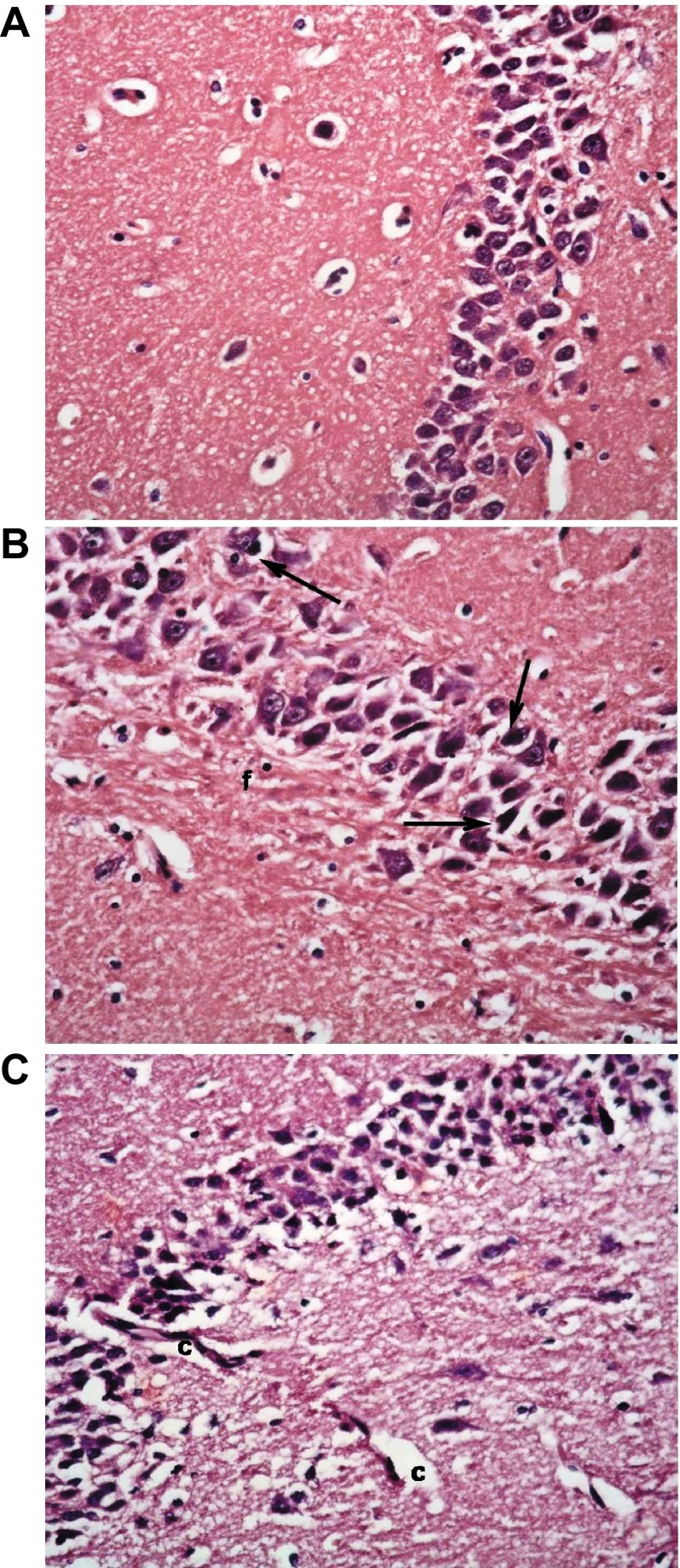
The type 1 group treated with insulin and memantine shows also ( **a**): preservation of small pyramidal cells of CA1 while; ( **b**): some of large pyramidal cells of CA3 show apoptosis (↑) with some clumping of neuronal fibrils (f) ( **c**): Granular cells show less vacuolation, & molecular layer shows normal size of cells, with widened capillaries ( **c**). (H & E ×400).

Addition of memantine to insulin therapy caused an additional mild improvement in the preservation of the cells, but with persistence of clumping of neuronal processes and widened capillaries in many fields ( Figure 11).

**Figure 12.**
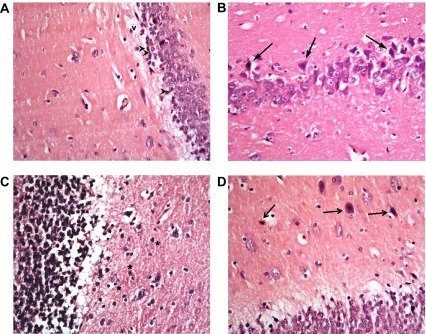
The untreated type 2 diabetic group shows changes similar to DM1. These include: ( **a**): many darkened nuclei of small pyramidal layer (arrowhead) mainly in deep layer, with vacuolation (v) and clumping of processes. ( **b**): layer of large pyramidal cells shows disorganization with many apoptotic cells (↑). ( **c**): granular layer shows some cell loss (arrowhead) and increased glial cells with no change in their size (*) while ( **d**) molecular layer shows apoptotic cells & (H & E ×400).

**Figure 13.**
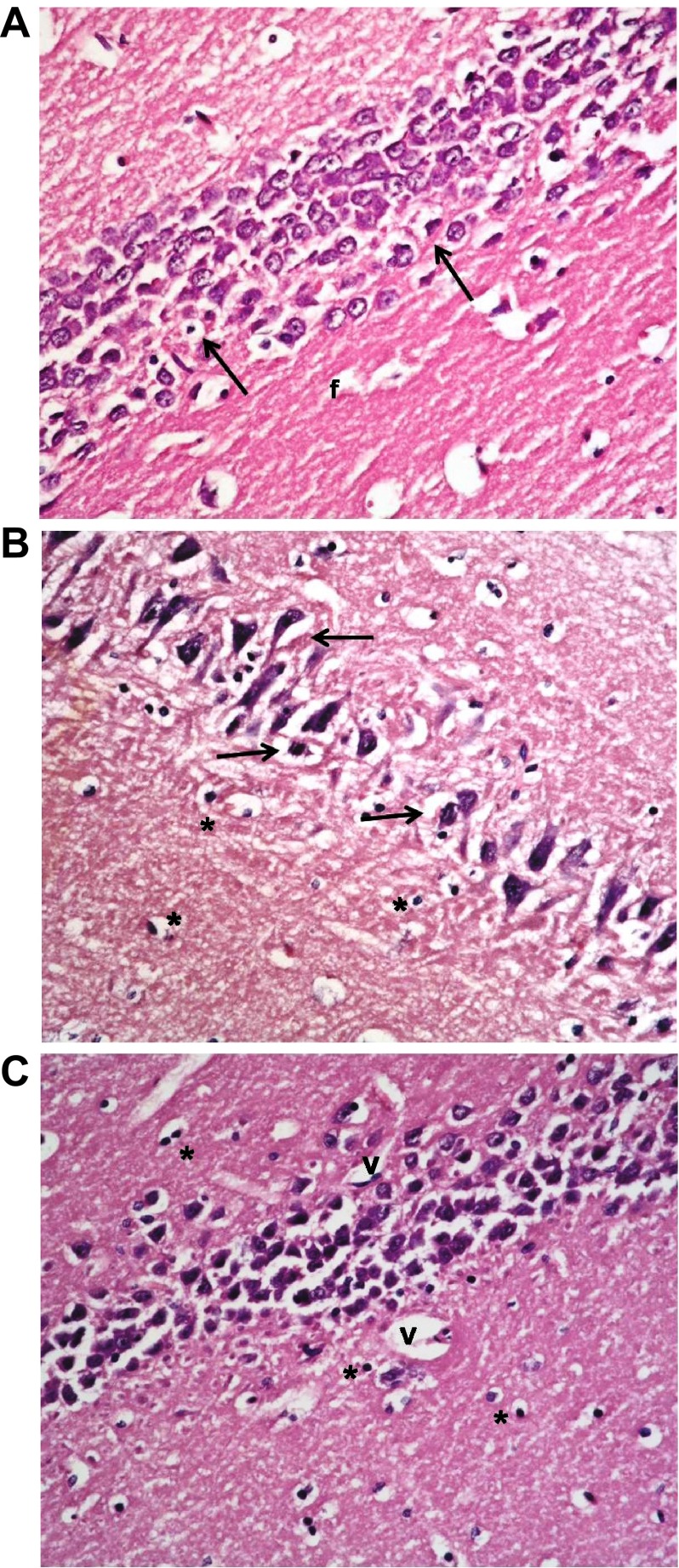
The type 2 diabetic group treated with memantine alone shows some protective effect for the drug in the form of: ( **a**): preservation of small pyramidal cells except for deepest layer (↑) with clumping of neuronal fibrils (f); but with ( **b**): shrinkage and darkening of many large pyramidal cells and ( **c**): clumping & disorganization of granular cells with dilated vessels (v) and normal glial cells (*) in molecular layer. (H & E ×400).

The type 2 DM group left untreated showed changes very similar to the type 1 untreated group. These included darkened small pyramidal cells mainly in deep part (inner layer nearer to the molecular layer), disorganization of layers and many apoptotic large cells, together with vacuolations and clumped processes, but with no significant change in the sizes of glial cells ( Figure 12). Treatment with memantine, however, caused a significant improvement in the form of fewer apoptotic cells, less marked shrinkage, but with persistence of clumped processes and dilated vessels ( Figure 13).

### Effect of diabetes and memantine on hippocampal synaptic markers

**Figure 14.**
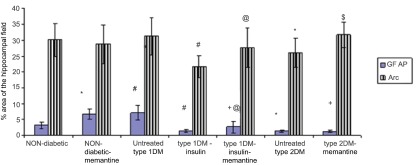
% area in the hippocampal field of glial fibrillatory acidic protein (GFAP) and Arc in the different studied groups assessed by immunohistochemistry. *: significant compared to non-diabetic group, +: significant compared to non-diabetic-memantine group, #: significant compared to untreated type 1 diabetic group, @: significant compared to type 1-insulin group, $: significant compared to untreated type 2 diabetic group at p<0.05. Data are presented as mean±SD (n=6/group).

As revealed in Figure 14, there was a highly significant increase in area % of GFAP staining of the non-diabetic-memantine group when compared to normal controls ( *p*<0.001). The untreated type 1 DM group also showed a very highly significant increase. Insulin treatment alone, however, caused a highly significant decrease in the level of staining that was just slightly better with the addition of memantine. The untreated type 2 DM group and memantine treated group both showed very low levels of staining, with a highly significant decrease when compared to control ( Figure 14).

There was no statistically significant difference in area % distribution of Arc between control, non-diabetic-memantine and untreated type 1 DM groups. The type 1 DM group receiving insulin had significantly decreased levels of staining as compared to controls. The type 1 DM group receiving insulin and memantine had significantly elevated levels when compared to the previous group, and were more similar to controls. The type 2 DM group exhibited a significantly lower level than the control group, which was also improved by the use of memantine ( Figure 14).

Treating the type 2 diabetic group with memantine significantly increased Arc compared to the untreated type 2 diabetic group ( Figure 14, *p*<0.05) and significantly decreased GFAP compared to the non-diabetic-memantine group. Administration of memantine to the non-diabetic group only significantly increased hippocampal GFAP expression compared to the non-diabetic group without affecting the hippocampal Arc.

## Immunohistochemical stains

### GFAP immunostaining

**Figure 15.**
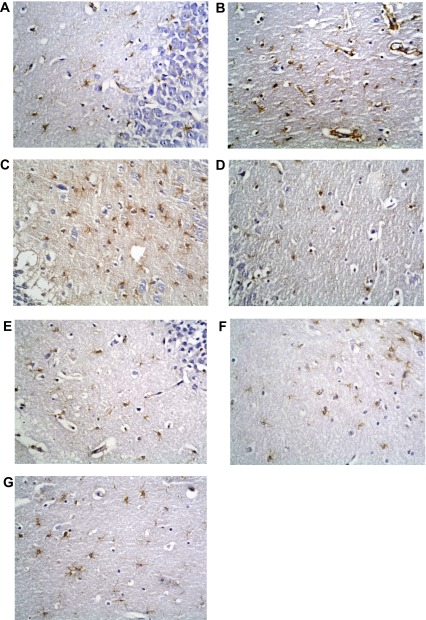
Anti-GFAP immunostaining x400. ( **a**) Non-diabetic control group. ( **b**) Non-diabetic memantine group. ( **c**) Type 1 DM untreated. ( **d**) Type 1 DM + insulin. ( **e**) Type 1 DM + insulin + memantine. ( **f**) Type 2 DM untreated. ( **g**) Type 2 DM + memantine. Note increased staining in b and c.

Immunohistochemical staining for GFAP showed its normal distribution in the control group as a mild positive reaction in glial cells of molecular layers. The non-diabetic group receiving memantine showed marked elevation in levels of immunostaining, becoming dense and more widespread in the enlarged glial cells. The type 1 DM untreated group also exhibited markedly high levels of reaction. However, this decreased to less than normal levels with insulin treatment alone, and was nearer to normal level with combined insulin and memantine therapy. The type 2 DM untreated group had a very low level of staining, which was not improved with memantine therapy ( Figures 15a–15g).

### Arc immunostaining

**Figure 16.**
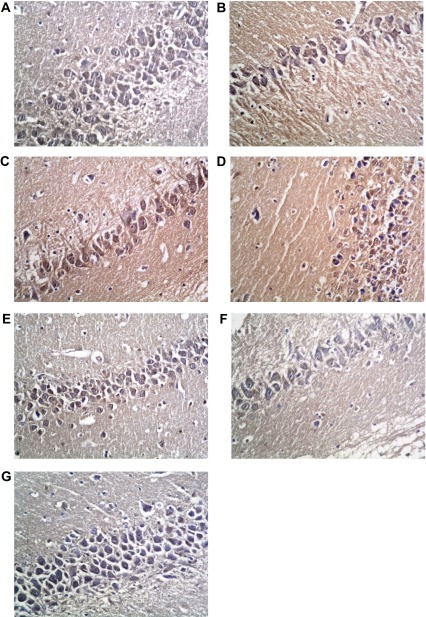
Anti-Arc immunostaining x400. ( **a**) Non-diabetic Control group. ( **b**) Non-diabetic memantine group. ( **c**) Type 1 DM untreated. ( **d**) Type 1 DM + insulin. ( **e**) Type 1 DM + insulin + memantine. ( **f**) Type 2 DM untreated. ( **g**) Type 2 DM + memantine. Note patchy disorganized staining in d, and to a lesser extent in e.

Immunohistochemical staining for Arc in the control group showed mild to moderate staining homogenously filling neuronal cell bodies, and widespread in neuronal processes. The level and distribution of staining was mostly not altered with memantine alone, or with type 1 DM alone. However, the type 1 DM group receiving insulin showed a significantly decreased level and disruption of normal homogenous patterns of staining in cell bodies. That returned to a near normal pattern and level with the addition of memantine therapy. The type 2 DM group exhibited only a mild level of widespread immunostaining that was significantly improved to a near-normal pattern with memantine therapy ( Figures 16a–16g).

## Discussion

Diabetes is associated with several adverse effects on the brain, some of which may result primarily from direct consequences of chronic hyperglycemia. In this work, both type 1 and 2 untreated diabetic groups showed significantly impaired learning and spatial memory in rats during the passive avoidance and Morris water maze tests compared to the non-diabetic group in agreement with previous studies ^1, 33^.

Insulin treatment of type 1 diabetic group after development of cognitive dysfunction partially improved cognitive functions compared to the untreated type 1 group; however, all tested cognitive functions were still impaired compared to the non-diabetic group. These results strongly support a previous study suggesting that intervention with insulin failed to reverse water maze learning and partially affected long-term potentiation, unlike insulin treatment commenced at the onset of diabetes ^34^.

Cognitive dysfunction and impaired synaptic plasticity in both types of diabetes have been linked to hyperglycemia, insulin deficiency and/or insulin resistance and altered insulin signaling ^35, 36^, hypophyseal-pituitary axis hyperactivity and elevated glucocorticoid levels ^37^. Insulin diminishes hypothalamic-pituitary-adrenal-axis activity ^38^ and modulates neurotransmitter levels ^39^, thus promoting physiologic processes critical for memory.

Hyperglycemia increases the NMDA receptor-mediated calcium entry into the neurons and may induce neuronal excitotoxicity through an activation cascade ending by the release of ROS ^40^. Memantine, an uncompetitive NMDA receptor antagonist, significantly improved serum glucose and insulin levels as well as HOMA-IR in type 1 and type 2 diabetic groups compared to the insulin-treated type 1 and untreated type 2 group, respectively. It also significantly improved all tested cognitive functions in the type 1 DM group (with the exception of the escape latency to hidden platform in the first three training blocks) compared to the type 1 group treated with insulin alone and in the type 2 DM group compared to the untreated type 2 diabetic group which indicates a positive effect of blocking NMDA receptors on memory and synaptic plasticity in diabetes. A similar dose of memantine has been shown to improve cognitive function and to slow cognitive and functional decline in Alzheimer disease transgenic rat models ^20^.

The importance of maintaining normal synaptic NMDA signaling was demonstrated by Papadia *et al.* ^41^. Memantine cannot act or accumulate in the NMDA channel when the channel is open for several milliseconds as occurs during normal synaptic activity. Instead, memantine only inhibits the prolonged influx of Ca ^2+^ ions and blocks abnormal glutamate excitatory signals ^42^.

Hippocampal formation has long been known to be responsible for learning and memory. These processes involve distinct, and interlinked, groups of efferent systems for episodic memory, affective and social learning, and sensory processing and integration ^43, 44^, all of which could be affected by chronic diseases like diabetes.

Examination of normal and diabetic hippocampus sections revealed marked effects of diabetes in the form of cell death in several areas, with disruption of normal layer organization. This was associated by clumping of neuronal processes (appearing as excess eosinophilia) which is indicative of damage to neurons ^45^. These changes were markedly improved by insulin therapy and by insulin and memantine, and were less improved by memantine therapy alone.

The reaction of astrocytes may be the earliest response of the brain tissue to an altered glucose metabolism ^46^. In this work, GFAP was significantly decreased in the untreated type 2 diabetic group compared to the non-diabetic group, in agreement with several previous studies ^12, 47^. However, GFAP was significantly elevated in the untreated type 1 diabetic group compared to non-diabetics, supported by the findings of Baydas *et al.* ^11^. These findings showed that untreated type 1 diabetes induced glial hyperactivity with increased GFAP in the rats' hippocampus. Baydas and colleagues attributed this to the effect of reactive oxygen species especially because oxidative stress occurs earlier in type 1 diabetes ^48^.

Moreover; Lebed *et al.* ^46^, assessed GFAP 3 and 7 days after STZ injection using immunohistochemistry and demonstrated that the reduced GFAP-positive cell count was found on day 3 when these cells were significantly smaller and less arborized with respect to the control. This tendency reversed on day 7 when more numerous GFAP-positive cells grew in size and became more ramified.

Insulin in the type 1 group significantly decreased hippocampal GFAP compared to untreated type 1 and non-diabetic groups. These results agree with those of Lechuga-Sancho *et al.* ^49^, but contradict those of Coleman *et al.* ^50^. Meanwhile, memantine significantly increased GFAP expression compared to the insulin-treated group, although it was still significantly lower than that of the non-diabetic-memantine group, and GFAP was changed insignificantly in the type 2 diabetic group compared to the untreated type 2 group.

The significant decrease in GFAP expression found in the current study in diabetic groups treated with memantine agrees with previous work by Miguel-Hidalgo *et al.* ^51^, who studied the neuroprotective effect of memantine against β-amyloid-induced neurodegeneration and concluded that memantine-treated animals had significant reductions in GFAP immunostaining as compared with vehicle-treated animals. Decreases in GFAP expression are associated with detrimental conditions in the CNS ^52^, several of which occur in GFAP knock-out mice and in diabetic individuals. Also; associated proliferation of astrocytic cells in the damaged area of hippocampus (increased GFAP immunostaining) is a common end result of damage to neurons in the CNS ^53^. In support of the cognitive dysfunction, Arc was significantly decreased in the untreated type 2 DM group compared to control. However; Arc expression was insignificantly changed in the untreated type 1 diabetic group compared to the control group. Both synaptic plasticity and memory are impaired in the absence of Arc ^13^, and inhibition of Arc protein in rats impaired maintenance of LTP beyond 4 hours ^54^.

While insulin in the type 1 group significantly decreased hippocampal Arc compared to the untreated and non-diabetic groups, memantine significantly increased its expression in the type 1 group compared to the insulin treated group, and in the type 2 diabetic group compared to untreated type 2 group making Arc expression nearer however still not identical to control group. Rosi *et al.* ^55^ found that memantine restored Arc in hippocampus CA3 cells that was disrupted by chronic neuroinflammation. Rial *et al.* ^56^ demonstrated that Arc reduces the number of glutamate receptors, leading to a decrease in α-amino-3-hydroxy-5-methyl-4-isoxazole propionic acid receptor (AMPAR) mediated synaptic currents, consistent with a role in the homeostatic regulation of synaptic strength.

In the present study, although Arc was insignificantly changed in the untreated type 1 diabetic group compared to the non-diabetic group, the type 2 diabetic groups showed a significant decrease in Arc expression in untreated type 2 compared to control. Mateos *et al.* ^57^, found decreased expression of Arc in the cerebral cortex and hippocampus of HFD-fed animals and linked this finding to HFD supplied to type 2 diabetic rats.

Since desensitization of insulin receptors and impaired insulin signaling are common features of diabetes ^58^, memantine may qualify as potential future option to combat cognitive impairments and dementia in diabetes.

The positive effects of memantine were absent in the non-diabetic group. Memantine significantly impaired all tested cognitive performances and significantly increased serum glucose. However, it did significantly increase hippocampal GFAP compared to the non-diabetic group, indicating that the beneficial effects of memantine are only evident in the disease state.

In conclusion, both types of diabetes are associated with cognitive dysfunction. Insulin in type 1 DM and memantine in both diabetic types could improve these parameters. Memantine had an advantage to be able to improve astrocytic reactivity in type 1 DM and Arc expression in both types of diabetes, which may spark a future therapeutic role of the NMDARs antagonists in diabetes-associated cognitive dysfunction.
