# Supplementary material for: A histological and functional study on hippocampal formation of normal and diabetic rats
Source: F1000Res. 2013 Jul 9;2:151. [Version 1] doi: 10.12688/f1000research.2-151.v1 (PMC3901513; doi:10.12688/f1000research.2-151.v1)
Supplement: Peer review history

# Peer review: Referee response for version 1

**Published:** 2013-07-17

**Authors:** Louiza Belkacemi

**Affiliations:** 1 The Life Science Health Center, University of Western Ontario, London, ON, Canada

This article sought to investigate the effect of diabetes mellitus on cognitive functions and related markers of hippocampal synaptic plasticity, and the potential impact of blocking N-methyl-D-aspartic acid (NMDA) receptors by memantine in type 1 and 2 diabetes mellitus in rat models. Their results showed that both types of diabetes negatively impact cognitive functions. 

The study is well designed and easy to follow but further clarifications are needed on the following points. 

**METHODS**

**Experimental design and animals**

- Explain why the albino rat was specifically chosen for their research.
- Explain how the 6 rats per group were chosen. Was power of analysis performed? 

**I** **nduction of diabetes**

- For the type 1 diabetes induced rats, when the single intraperitoneal (i.p.) injection of 65 mg/kg STZ was given and for how long?
- For type 2 diabetes, why the investigators fed the rats high fat diet (HFD: 58% Kcal fat) for a period of 2 weeks before STZ injection? 
- How often the blood glucose levels and body weights of all rats were assessed? Did the investigators measure the blood glucose of all the rats before STZ injection of the diabetes type 2 DM groups? If so, how glucose levels of injected type 1DM, type 2 DM (before injection) compares to the controls, and how type 1 DM compares to type 2 DM (before injection)? Do the authors think that the fat diet may be a confounding parameter in the type 2 DM?

**Insulin treatment**

- Please add when exactly the insulin treatment was started for each group. Explain why the dose of insulin given to the treated rats was the same in type and type 2 diabetic rats.  Why the subcutaneous route was used for injection?

**Histological staining**

- The authors wrote that the brains were removed and placed in a new formaldehyde solution for 24 hrs. Please add the concentration of this paraformaldehyde solution. Were the brains left at room temperature or stored at 4°C during those extra 24 hrs prior to paraffin embedding?
- GFAP immunoreactivity scoring using QWin 500 software requires a more accurate description. For example, describe how immunoreactive areas were chosen? How areas were classified as positive or negative? How thresholds evaluating color and intensity of staining were selected?

**RESULTS**

**Metabolic parameters in the different studied groups**

- **How type 2 DM group treated with both insulin and memantine compares with type 2 treated with **insulin alone?
** **

**Hematoxylin and eosin stained sections**

- In figure 8, please give the percentage decrease in thickness of small and large pyramidal cell layers from non-diabetic groups receiving memantine alone. Also add the percentage of enlargement of pyramidal cells.
- In figure 9, please add the percentage of size decrease in the large pyramidal cells from the type 1 DM group?
- In figure 10, by how much the level of apoptosis in large cells was decreased? Describe how the number of apoptotic cells was counted?
- In figure, 11, by how much the level of apoptosis in large cells was decreased in the group treated with memantine? And how dilation of vessels was evaluated?

**Effect of diabetes and memantine on hippocampal synaptic markers**

- Give % ARC distribution for all experimental conditions vs. respective matched controls.
- Give the % of GFAP staining for all experimental conditions vs. respective matched controls.

**IMMUNOHISTOCHEMICAL STAINS**

**GFAP immunostaining**

- The title of this section is confusing. What is the difference between area of Arc and GFAP distribution and immunostaining of these two proteins? Please clarify.
- Please add % level or fold change of GFAP and ARC immunostaining for type 1 DM and type 2 DM, as compared to their respective controls.
- All the figures should have scale bars, not just magnification at which the pictures were taken.

**DISCUSSION**

- The first sentence of this section “Diabetes is associated with several adverse effects on the brain, some of which may result primarily from direct consequences of chronic hyperglycemia” needs reference.
- The authors should discuss findings that contrast with theirs.

I have read this submission. I believe that I have an appropriate level of expertise to confirm that it is of an acceptable scientific standard, however I have significant reservations, as outlined above.

# Peer review: Referee response for version 1

**Published:** 2013-07-16

**Authors:** Zhenjie Zhang

**Affiliations:** 1 Stanford School of Medicine, Stanford University, CA, USA

This study by Amin *et al*. accessed the effects of memantine (NMDA receptor blocker marketed as an Alzheimer’s disease medication) on hippocampal histology and functions in diabetes model rats.  The authors presented interesting data.  Both of their functional and histological results showed severe impairment in pathological groups, and memantine partially alleviated these impairments in some cases.  However, in the non-diabetic group treated with memantine, impairments similar to the pathological groups were observed.  Thus, the potential benefit by memantine was only evident in diabetic conditions. While there are interesting observations in the study, it also raises concerns regarding the experiment design and data interpretation. Major concerns:

1. While the authors claimed memantine effectively improved nearly all quantitative test scores in both type I and type II diabetes groups, some improvement data upon the type I group were actually lacking.  The authors only compared the type I diabetic rats treated with insulin to type I diabetic rats treated with both insulin and memantine, but did not compare untreated type I diabetic rats to memantine treated type I diabetic rats.  Thus, based on the presented data, it is very difficult to access the effects of memantine alone on type I biabetic hippocampal function and histology.      
2. While the paper concludes that memantine significantly improved the type I diabetic pathology, the data presented showed only very marginal improvements by memantine on top of insulin (especially Figure 2, 4, 5).  These differences were statistically significant only because of the extremely small standard deviations in the data.

  Minor concerns:

1. Some wordings describing effects need to be changed.  Specifically, the last paragraph of page 7 says “Administration of memantine to the non-diabetic group had less beneficial effects…”  In fact, that treatment severely impaired all functions tested, instead of just being “less beneficial”. 
2. The SDs in many of the graphs are very small.  It would be a lot more informative if statistical significance levels of p<0.01 and p<0.001 were included in addition to the mere p<0.05.  This would help the readers to appreciate the statistical strength of the conclusions.

I have read this submission. I believe that I have an appropriate level of expertise to confirm that it is of an acceptable scientific standard, however I have significant reservations, as outlined above.

# Peer review: Referee response for version 1

**Published:** 2013-07-16

**Authors:** Carlos Telleria

**Affiliations:** 1 Division of Basic Biomedical Sciences, University of South Dakota School of Medicine, Vermillion, SD, USA

This article presents experiments that are very well described. The title is appropriate for the content of the manuscript and the conclusions are appropriate to the results obtained and not overstated. Materials and methods are clearly described so as the experiments can be replicated. The study could be strengthened by adding early on in the manuscript (e.g. introduction) more information about memantine, which is the NMDAR blocker used in the study.

I have read this submission. I believe that I have an appropriate level of expertise to confirm that it is of an acceptable scientific standard.
